# Discrepancies at clinical presentation of patients with soft tissue sarcoma according to the type of health insurance in a Brazilian population

**DOI:** 10.1371/journal.pone.0320308

**Published:** 2025-04-15

**Authors:** Fernando Augusto Batista Campos, Marcelo Porfírio Sunagua Aruquipa, Celso Silva e Sousa Filho, Maria Rita Silva Costa, Celso Abdon Lopes de Mello, Ulisses Ribaldo Nicolau, Suely Akiko Nakagawa, Antônio Geraldo do Nascimento, Maria Nirvana da Cruz Formiga, Felipe D’Almeida Costa, Maria Letícia Gobo Silva, Ademar Lopes, Samuel Aguiar Júnior

**Affiliations:** 1 Department of Medical Oncology, A.C.Camargo Cancer Center, São Paulo, SP, Brazil; 2 Department of Orthopedic Surgery, A.C.Camargo Cancer Center, São Paulo, SP, Brazil; 3 Department of Pathology, A.C.Camargo Cancer Center, São Paulo, SP, Brazil; 4 Department of Radiotherapy, A.C.Camargo Cancer Center, São Paulo, Brazil; 5 Department of Surgical Oncology, A.C.Camargo Cancer Center, São Paulo, SP, Brazil; FIOCRUZ: Fundacao Oswaldo Cruz, BRAZIL

## Abstract

**Background:**

In Brazil, 75% of the population is covered by public health insurance (PubHIn), and the rest pay for private insurance (PrivHIn). The impact of the type of health insurance on clinical presentation of Brazilian patients diagnosed with soft tissue sarcoma (STS) is unknown. We aim to describe the clinical characteristics at diagnosis of patients with STS, stratifying them by type of health insurance, and to evaluate the symptom-diagnosis and -treatment intervals.

**Methods:**

Observational, retrospective cohort, single-center study conducted in a Brazilian cancer center that admits both private and public patients. Medical records of individuals with 18 years old or more diagnosed with selected types of STS who started treatment in our center between January 2011 and December 2019 were reviewed. Kaplan–Meier method and log-rank test were used for survival analyses. Chi-square and Mann Whitney test were used for univariate analysis. Significance was defined as a p-value <0.05.

**Results:**

225 patients were included: 25.3% (n=57) with PubHIn and 74.7% (n=168) with PrivHIn. Median tumor size in PrivHIn was 7 cm (0.9–45 cm) versus 10.3 cm (0.8–30.5 cm) in PubHIn (p=0.0001). Metastases were present in 8.9% of patients with PrivHIn (n=15) and in 26.3% (n=15) of those with PubHIn (p=0.002). Median symptom-diagnosis interval was 195 days in the PubHIn group and 163 days in the PrivHIn group (p=0.74). For the whole series, median age at diagnosis was 51 years (18–85 yo). Lower limbs were the most frequent location of tumors (53.8%), and 86% had histological grade 2/3.

**Conclusions:**

Patients with STS covered by PubHIn presented larger tumors and more metastatic disease at diagnosis, suggesting that there may be disparities in health care delivery and limited access to health resources. A better understanding of the barriers in the journey of patients with STS could improve outcomes.

## Introduction

Soft tissue sarcomas (STS) comprise a heterogenous group of rare neoplasms with mesenchymal differentiation and represent about 1% of cancers [1]. Surgical resection with wide margins is the only curative therapy for localized disease [2]. Radiotherapy and chemotherapy can contribute to limb-sparing surgeries in addition to increasing local control and prolonging survival in selected cases, respectively [3]. Therefore, early diagnosis has a significant impact on delivering appropriate local treatment with the lowest morbidity possible while preserving functionality and quality of life [4].

The annual incidence of STS in Brazil is unknown. Recently, David et al. reported the first demographic data of Brazilian patients diagnosed with sarcoma extracted from the Brazilian Hospital–Based Cancer Registries System [5]. They showed that most of the individuals (~ 70%) were diagnosed with localized disease, and a significant part of the patients (~ 40%) initiated treatment more than 60 days after diagnosis.

In Brazil, 75% of the population is covered by the Brazilian Unified Health System (Sistema Único de Saúde − SUS), a public health insurance (PubHIn) with universal health scheme, and 25% pay for a private health insurance (PrivHIn) [6]. The general disparities between these two systems are striking. Oncological patients with PubHIn face long waiting times on their journey until the start of treatment caused by restricted access to the basic health system, the absence of a structured referral network of specialized units, a small number of specialized professionals, and difficulty in accessing diagnostic procedures and pathology services, among others, which negatively impact the staging of disease at presentation and the clinical outcomes [7]. For instance, in a study by the Brazilian Group of Breast Cancer, it was shown that around 39% of women with breast cancer covered by PubHIn are diagnosed in stages III-IV, whereas in those with PrivHIn it is about 20% (p < 0.001) [8]. There is no data on the impact of health insurance on the staging of Brazilian patients with sarcoma, or on the interval between the first symptom to diagnosis, which is essential for the improvement of health policies, specifically for patients covered by the PubHIn, that is, most of the Brazilian population.

Therefore, we aimed to characterize the clinical staging of patients with STS treated in a Brazilian cancer center that treats both patients with PrivHIn and PubHIn, and to evaluate the symptom-to-diagnosis and symptom-to-treatment intervals comparing findings between the PrivHIn and PubHIn systems.

## Methods

### Patients

**Fig 1:**
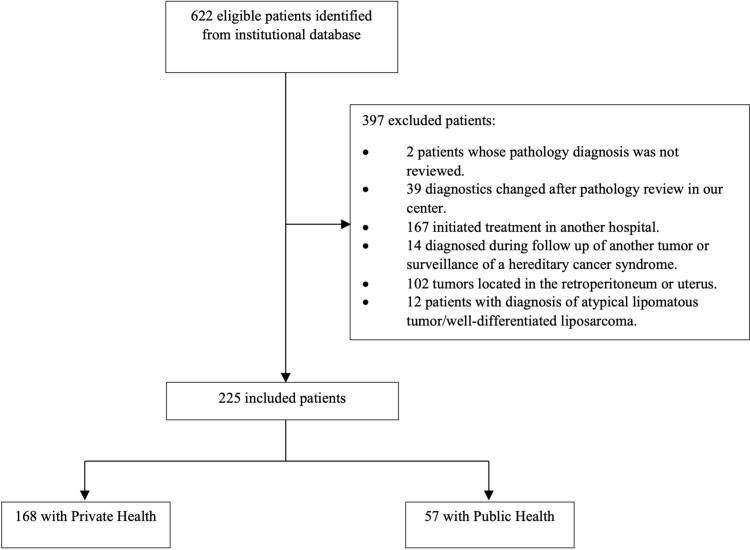
Flow diagram showing the process of selecting patients.

This is an observational, retrospective, single-center study conducted at A.C.Camargo Cancer Center, São Paulo, SP, Brazil. Medical records of patients with 18 years old or more diagnosed with soft tissue sarcoma who started treatment in our center between January 2011 and December 2019 were reviewed. We included only patients whose pathological diagnosis was made or reviewed by one of our sarcoma pathologists by the time of diagnosis (Fig 1). The following histologies were included: leiomyosarcoma (LMS), liposarcoma, undifferentiated pleomorphic sarcoma (UPS), synovial sarcoma (SS), malignant peripheral nerve sheath tumor, and myxofibrosarcoma. The exclusion criteria were: (1) patients who started treatment at another hospital, (2) patients diagnosed during follow-up of another type of malignancy, (3) patients diagnosed with a sarcoma during screening images due to a known hereditary cancer syndrome, (4) patients with primary tumors located in the retroperitoneum or uterus, (5) patients diagnosed with well-differentiated liposarcoma/atypical lipomatous tumor.

To characterize the clinical staging of the patients at diagnosis, the variables collected were tumor size, the presence of lymph node, and distant metastasis. We also collected information about potentially confounding variables such as age, sex, pathological subtype, tumor grade, and tumor location to confirm that the cohorts were balanced.

Data were gathered from electronic clinical charts, and a retrospective database was constructed for analysis. Medical records were accessed between 07/01/2022 and 01/31/2023 for this research purposes. All collected data were anonymous. This study was conducted after approval from the Fundação Antônio Prudente - A.C.Camargo Cancer Center Ethics Committee, certified by the Brazilian National Research Ethics Commission (number 5432); study registration number 3059/21, approval number 4.758.257. Due to the retrospective observational design of this study, and since we used anonymized data, informed consent was waived by the Fundação Antônio Prudente - A.C.Camargo Cancer Center Ethics Committee. All methods were carried out in accordance with relevant guidelines and regulations.

### End point definition

The date of diagnosis was defined as the date of biopsy-proven disease. Symptom-to-diagnosis interval was defined as the time between the first symptom referred by the patient and the histopathological diagnosis at our institution; symptom-to-treatment interval was defined as the time between the first symptom and treatment initiation (surgery, radiotherapy, or chemotherapy). Both intervals were measured in days. Overall survival (OS) was calculated from the date of diagnosis to the date of death from any cause.

### Statistical analysis

Distribution of frequency was used to describe the categorical variables with relative and absolute values, and the numerical variables were described with median values. Survivals were estimated by the Kaplan-Meier method, and the log-rank test was used to compare the groups. Patients who did not die during the study period were censored at their most recent follow-up at the institution. To compare categorical and continuous variables, we used the chi-square test or Fisher’s exact test and Mann Whitney, respectively. The Shapiro-Wilk test was used to assess normality. Statistical significance was defined as a p-value <0.05. Statistical analyses were performed on SPSS version 23.

## Results

Two hundred twenty-five patients meeting the inclusion/exclusion criteria were included in the final analysis: 168 (74.7%) with PrivHIn and 57 (26.3%) with PubHIn. The clinicopathological characteristics and comparisons between findings are shown in Table 1. Median age at diagnosis was 51 years (18–85 years). The majority of the individuals were < 60 years old (69.8%) and were female (51.6%). The most frequent histological subtypes were LMS (23.1%), UPS (20.4%), and SS (19.1%). At diagnosis, the median tumor size of the patients from the private system was 7 cm (0.9–45 cm) versus 10.3 cm (0.8–30.5 cm) from those in the public system (p = 0.0001). The primary site of disease was the lower limbs in 53.8% of patients, and 85.8% of the tumors showed histological grade 2 or 3. Metastases were present at diagnosis in 15 patients (8.9%) with PrivHIn and in 15 patients (26.3%) with PubHIn (p = 0.002).

**Table 1:** Clinicopathological characteristics of the patients included in the study and univariate analysis.

Variable	Total	PrivHIn	PubHIn	*p* value
	n = 225 (100%)	n = 168 (74.7%)	n = 57 (25.3%)	
Age (years), median (range)	51 (18–85)	52 (20–85)	48 (18–85)	0.566
Sex, n (%)				–
Male	109 (48.4)	82 (46.9)	35 (56.5)	
Female	116 (51.6)	93 (53.1)	27 (43.5)	
Histology, n (%)				0.278
Liposarcoma	60 (26.7)	44 (26.2)	21 (33.9)	
Myxoid Liposarcoma	33 (14.7)	24 (14.3)	9 (15.8)	
DDLPS	18 (8.0)	11 (6.5)	7 (12.3)	
Pleomorphic Liposarcoma	9 (4.0)	9 (5.4)	0 (0)	
Leiomyosarcoma	52 (23.1)	41 (24.4)	11 (19.3)	
UPS	46 (20.4)	34 (20.2)	12 (21.1)	
Synovial Sarcoma	43 (19.1)	30 (17.9)	13 (22.8)	
Myxofibrosarcoma	20 (8.9)	17 (10.1)	3 (5.3)	
MPNST	4 (1.8)	2 (1.2)	2 (3.5)	
Tumor size (cm), median (range)	7.9 (0.8–45)	7 (0.9–45)	10.3 (0.8–30.5)	0.0001
Histological grade, n (%)				1.0
1	29 (12.9)	22 (13.2)	7 (12.7)	
2 or 3	193 (85.8)	145 (86.8)	48 (87.3)	
Not informed	3 (1.3)	1 (0.6)	2 (3.5)	
Location, n (%)				0.734
Lower extremity	121 (53.8)	89 (53.0)	32 (56.1)	
Upper extremity	31 (13.8)	25 (14.9)	6 (10.5)	
Trunk wall	28 (12.4)	19 (11.3)	9 (15.8)	
Abdominopelvic	27 (12.0)	21 (12.5)	6 (10.5)	
Head and neck	16 (7.1)	13 (7.7)	3 (5.3)	
Other	2 (0.9)	1 (0.6)	1 (1.8)	
Lymphadenopathy, n (%)				1.0
Yes	7 (3.1)	2 (3.5)	5 (3.0)	
No	218 (96.9)	55 (96.5)	163 (97.0)	
Metastasis, n (%)				0.002
Yes	30 (13.3)	15 (8.9)	15 (26.3)	
No	195 (86.7)	153 (91.1)	42 (73.7)	

PrivHIn: private health insurance; PubHIn: public health insurance; DDLPS: dedifferentiated liposarcoma; UPS: undifferentiated pleomorphic sarcoma; MPNST: malignant peripheral nerve sheath tumor.

The median time interval between the onset of symptoms and pathological diagnosis for the entire population was 180 days (7–2182). Symptom-diagnosis interval was 163 days for patients with PrivHIn and 195 days for those with PubHIn (p = 0.748), and the median time interval to treatment initiation was 198 days in PrivHIn group and 231 days in PubHIn group (p = 0.709). Although also not statistically significant, female patients and those < 60 years had a larger interval in comparison to men and patients > 60 years, respectively (Table 2).

**Table 2:** Symptom-diagnosis interval in different groups of patients and comparison by Mann-Whitney U test.

	Symptom-diagnosis interval (days), median (range)	*p* value
Study population	180 (7–2182)	–
PrivHIn	163 (7–2182)	0.748
PubHIn	195 (35–760)	
< 60 years	183 (7–1489)	0.586
> 60 years	162 (19–2182)	
Female	184 (7–1467)	0.224
Male	158 (19–2182)	

**Fig 2:**
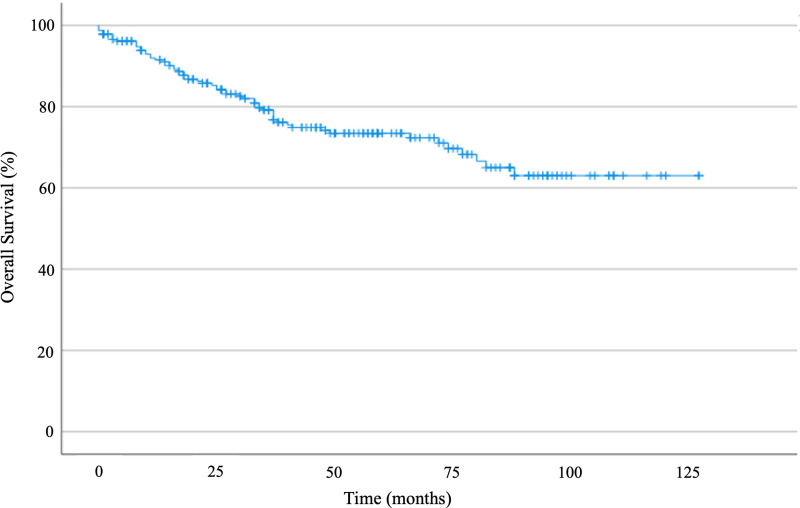
Kaplan-Meier curve showing overall survival for the entire study population.

**Fig 3:**
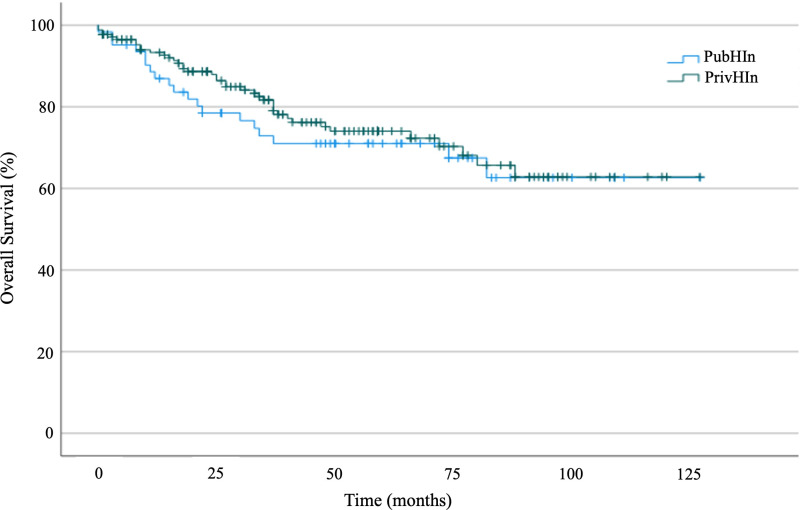
Kaplan-Meier curve showing overall survival for patients with private health insurance (PrivHIn) and public health insurance (PubHIn).

The median follow-up time was 57 months (95% CI 53.2–60.8), and the median OS was not reached (Fig 2). For patients with metastases at diagnosis, median OS was 27 months (95% CI 9.7–44.2). Five-year OS was 72% in the study population, with no statistically significant difference between PrivHIn and PubHIn (73% versus 68%, p = 0.418 – Fig 3). In the univariate analysis, factors related to worse OS were high histological grade, size of lesion, metastasis at diagnosis, and histological subtype. No survival correlation was found for symptom-diagnosis and symptom-treatment intervals.

Regarding the correlation between delay of diagnosis and size of the lesion, the Pearson correlation test in our study was positive in the entire population, although weak (0.180, p < 0.001). When stratified by histological grade, the significance was lost for grade 1 tumors (0.316, p = 0.132), while it was kept for high-grade lesions (0.166, p = 0.029). The Mann-Whitney test to correlate longer symptom-diagnosis intervals with the presence of metastasis at diagnosis was not significant (p = 0.148) in the total population. When stratified by histological grade, no difference was found for grade 1 tumors, whereas in grade 2/3 STS a numerical difference was seen (5.3 months for non-metastatic patients versus 9.8 months for patients with metastasis at diagnosis, p = 0.055).

## Discussion

There are few studies evaluating the epidemiological profile of STS in Brazil. Herein, we assessed the clinical presentation and the prognosis of patients with STS treated in a Brazilian cancer reference center, stratifying them by type of medical health coverage. Our results show a significant and worrisome discrepancy in the disease staging at presentation: patients with PubHIn presented with larger lesions and more metastatic disease in comparison to those with PrivHIn. To our knowledge, this is the first study analyzing the impact of medical health coverage on presentation of patients with STS in Brazil and the intervals between symptoms onset and diagnosis.

Data showing the impact of insurance on the staging of STS is scarce in the literature. Most studies were performed in the United States, where it was found that disparities in insurance status were associated with an increased risk for metastatic disease at diagnosis [9,10]. For instance, the study by Smartt et al. reporting findings from the Surveillance, Epidemiology, and End Results database (SEER) showed that patients with extremity STS with Medicaid were more likely to present stage IV disease in comparison to those with non-Medicaid insurance (11% versus 4.9%; relative risk, 2.4 [95% CI 1.9 to 3.1]; p < 0.001) [10]. In the United States, Medicaid is the government program of health coverage for low-income individuals and secures 18.9% of the population [11].

The rarity of STS and the wide variability in clinical presentation explain the inexperience of health professionals outside specialized centers in recognizing these tumors, which leads to incorrect diagnoses and a delay in proper diagnosis [12]. Data from a survey made in the United Kingdom indicate that patients with sarcoma, unlike those diagnosed with other types of malignancy, have more appointments at primary care services, and that about 30% of them wait at least 6 months before reaching a diagnostic conclusion [4]. This is enhanced when patients are managed in public health systems in developing countries, in which, specifically in the case of Brazil, many obstacles are faced by people during the medical journey, such as the distance of the facilities from their home, low social or familiar support, few primary care physicians, and a lack of integrated care protocols based on specialized medical guidelines [13,14].

On the other hand, patients assume a notable contribution to the delay in diagnosis. There is a tendency to underestimate the symptoms, and the presence of a nodule or mass, the main signal of the disease, is often attributed to previous trauma or insect bites or stings. In addition, the insidious and non-painful nature of the lesions, with little impact on quality of life, leads the person to take a long time to seek health services [15,16]. In the case of the Brazilian public health system, it cannot be overlooked that the difficulty in accessing high quality health services affects the population’s decision to seek them. This is added to the lower socio-cultural level of the population covered by the PubHIn, which also contributes to more advanced disease in this group [14,17].

It is important to highlight that although approximately 75% of Brazilians rely on PubHIn for healthcare, only 25% of the patients in our study were covered by PubHIn, which introduces a significant bias, especially regarding OS estimates. Several factors may explain this discrepancy. First, because Brazil’s public healthcare system lacks a specialized sarcoma network, many PubHIn patients undergo initial treatments outside specialized cancer centers, leading to their exclusion from our analysis. Additionally, over the past decade, our institution has experienced a decline in referrals from PubHIn due to revisions of contracts with the municipal public health system. Since more recent patient records are typically more complete, they were more likely to be included in the study, further contributing to the underrepresentation of PubHIn patients.

We evaluated the symptom-diagnosis interval in our population, finding a median of 180 days (163 days in the PrivHIn and 195 days in the PubHIn, p = 0.74). This result is in line with the findings of most other studies from different countries (Table 3). Currently, there is still no established consensus on a cut-off point that discriminates between a short and a long symptom-diagnosis interval for STS or whether this has any impact on overall survival [18]. It is more likely that the delay in diagnosis is associated with larger tumor diameters, an increased risk of amputations, worse functional outcomes, and also a negative impact on patient fertility, psychological distress, patient dissatisfaction, and low adherence to treatment [15,19,20]. All together, this leads to a higher cost of health care and a rise in judicialization processes to obtain off-label treatments [21].

**Table 3:** Retrospective studies reporting the symptom-diagnosis interval in patients with soft tissue sarcomas.

Author, year	Number of patients	Country	Median symptom-diagnosis interval (days)
Brouns, 2003 [12]	100	Belgium	179
Clark, 2005 [22]	159	United Kingdom	420
Park, 2010 [23]	18	South Korea	360
Nakamura, 2011 [20]	100	Japan	180
Goedhart, 2016 [24]	54	Netherlands	160
Dyrop, 2016 [19]	102	Denmark	176

Despite the finding of more advanced disease at diagnosis in the PubHIn group, there was no statistical difference in OS in our analysis. An American study including pediatric and adult patients, with a third of the cohort having bone sarcomas, observed a significant difference in the 5-year OS between patients treated in the PubHIn and PrivHIn systems (61 vs. 71%, respectively, p = 0.001) [25]. On the other hand, another retrospective study of the same region, but with a smaller population, also including about a third of bone sarcomas, observed larger tumors at presentation with a longer delay to diagnosis for patients with PubHIn, but without statistical difference in the OS between the 2 types of health insurance [26]. This lack of consistency in the results of the series, even from the same region, could be explained by the heterogeneity of the STS and the different expertise between medical facilities [2].^2^ In our study, some factors may explain the lack of differences in OS between patients in the PrivHIn and PubHIn groups. First, patients in our study were managed at an expert sarcoma center. More importantly, as previously mentioned, a selection bias in the PubHIn is evident, which may have influenced the OS analysis.

Our clinical data is compatible with other studies from Latin America referring to median age at diagnosis (about 50 years) and histological subtypes frequency. For instance, in the largest study including patients with extremity STS from a single referral center in Latin America, García-Ortega et al. reported a median age at diagnosis of 45 years (15–95 years) and a prevalence of liposarcomas, which is quite the same finding in a study from the National Institute of Neoplastic Diseases in Peru (mean age of 52.6 years, with 69.6% of patients older than 50 years; and liposarcomas as the second most common histology) [27,28]. Despite this, it must be noted that clinical characteristics such as tumor location, size, histological grade, and metastasis are difficult to compare with other studies because of the rarity and heterogeneity of the STS, different inclusion criteria, as well as different access to diagnostic tests such as immunohistochemistry, added to the sub-notification of cases in many regions with difficult access to medical centers [5,29].

Our analysis showed a numerically larger interval from the onset of symptoms and pathological diagnosis as well as the start of treatment for patients with PubHIn compared to those with PrivHIn, but this difference was not statistically significant in the chi-square test. This finding could be related to the subjectivity of the onset of symptoms reported by patients, the different socio-economic realities around Brazilian regions, and the reduced number of participants from only one center. The reference system in Brazil has not developed a specialist-oriented network, and patients are referred to the nearest oncology service from their residence, not necessarily a sarcoma reference center. This happens mainly because health administrators are pushed by civil organizations to maintain the time from the diagnosis to the first oncology consultation below 60 days, a Brazilian law called “the 60-day” policy [30].

Other limitations of our study were the fact that it was performed with a single-center database, the lack of description of other clinical information in medical records such as comorbidities, socioeconomic status, and access to medical facilities. It is important to note the retrospective nature of our study, which is subject to biases in information and interpretation by the physicians when registering the medical records. In addition, it must be highlighted that Brazil is a nation with great inequality in terms of access to and quality of the health system, both for the public and private systems [31]. In this context, it is likely that patients relying on PubHIn remain undiagnosed due to both low levels of education and limited access to an adequate healthcare system capable of timely diagnosis. Our center is located in the most developed region of the country. It is quite probable that the differences found in our study are much more pronounced in the other regions of Brazil, which might impact the survival outcomes.

## Conclusion

In our cohort, patients with STS covered by PubHIn presented significantly larger lesions and a higher chance of having metastatic disease at diagnosis than those with PrivHIn. Time from symptom to diagnosis and treatment was longer in PubHIn, without statistical significance. These findings show the long journey from symptoms to treatment, suggesting low expertise in sarcoma diagnosis and management among health services in Brazil, as well as low awareness of the disease among the population.

Further studies are needed to understand the journey of patients with sarcoma through the Brazilian health system, both public and private, and the risk factors for a longer delay until diagnosis. This understanding is essential for the development of health policies capable of impacting clinical outcomes and for the rational use of health resources.
